# Impact of Chronic Pain on Anxiety, Depression, and Daily Life Activities Among Breast Cancer Survivors in Tunisia

**DOI:** 10.1192/j.eurpsy.2025.1778

**Published:** 2025-08-26

**Authors:** A. Hadj Salah, M. Chaouat, A. Haouala, A. Krir, S. Zaied, F. Zaafrane, A. Mhalla, B. Amamou

**Affiliations:** 1 psychiatry; 2 oncology, Fattouma Bourguiba Hospital, Monastir, Tunisia

## Abstract

**Introduction:**

Chronic pain is a prevalent issue among breast cancer survivors, often causing significant distress, disability, and interference with daily activities. Affecting around 18% of the global population, chronic pain is the leading cause of years lived with disability. Among breast cancer survivors, it has become a key focus due to its high prevalence and impact on quality of life.

**Objectives:**

To assess the prevalence and characteristics of chronic pain in breast cancer survivors and examine its correlation with anxiety, depression, and interference with daily life activities.

**Methods:**

This is a cross-sectional descriptive and analytic study conducted on 100 women treated for breast cancer at the medical oncology department of Fattouma Bourguiba University Hospital. The study spanned eight months (June 2021–February 2022). Pain characteristics were assessed, and anxiety and depression levels were measured using the Hospital Anxiety and Depression Scale (HADS). Pain-related catastrophic thinking was evaluated with the Pain Catastrophizing Scale (PCS), and family functionality was measured using the Family APGAR score.

**Results:**

The study involved 100 breast cancer survivors, with a mean age of 53.6 ± 10.1 years.

Our entire population confirmed having chronic pain for more than 3 months, most of them (53%) had it from 2 to 5 years

The median duration of chronic pain was 3 years [IQR: 2-5], with the most common pain locations being the breast area (72%) and upper limb (27%). Pain intensity was predominantly mild (62%), followed by moderate (34%), and severe (4%).

Chronic pain significantly interfered with daily activities, especially in patients with moderate to high pain intensity (p < 0.001), affecting both affective and activity clusters. The mean Pain Catastrophizing Scale score was 7.79 ± 5.25, indicating mild catastrophic thinking, which was most likely due to the low pain intensity experienced by the majority.

Anxiety levels were significantly correlated with higher pain intensity (p = 0.013), whereas depression, though elevated in patients with more severe pain, did not demonstrate a statistically significant association (p = 0.135).

The majority of participants were from highly functional families, with 88% reporting strong family support

Despite the prevalence of chronic pain, fatigue was not significantly related to pain intensity.

**Conclusions:**

Chronic pain in breast cancer survivors significantly interferes with daily activities, particularly among those with moderate to high pain intensity. Anxiety is notably more prevalent in patients experiencing more severe pain. These findings underline the importance of comprehensive pain management strategies and psychological support for breast cancer survivors.

**Disclosure of Interest:**

None Declared

